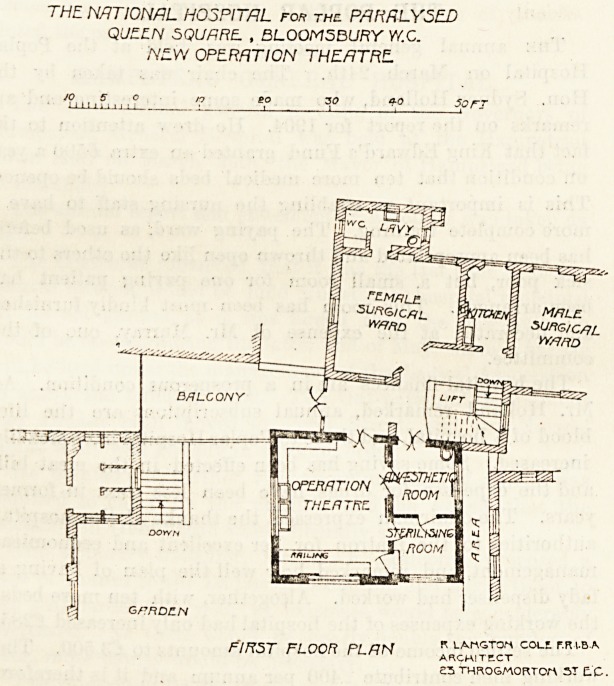# New Operation Theatre at the National Hospital for the Paralysed and Epileptic

**Published:** 1905-04-08

**Authors:** 


### NEW OPERATION THEATRE AT THE NATIONAL HOSPITAL FOR THE PARALYSED AND EPILEPTIC.

In a late issue of The Hospital we referred to this theatre; and we are now able, by the courtesy of the architect, to publish the plans. The theatre is on the first floor of the hospital, and in close proximity to the surgical wards and to the staircase and lift. It projects into the garden of the hospital, or rather it has been raised on piers placed therein by which means a shelter under the theatre has been obtained, and an improvement effected to the garden. The new block is about 30 feet by 20 feet, and it consists of an anæsthetic-room, a sterilising-room, and the theatre proper. The latter is about 20 feet by 15 feet, and a small part of it has been railed off presumably for onlookers during the operations. All the rooms open into each other, and the anæsthetic-room and also the theatre have doors opening on the corridor. All the arrangements are simple and good. An electric fan is used as an air extractor; and sterilised water is supplied by a Berkenfeld filter.

The cost complete was £1,200—a very moderate outlay indeed; and when we state that the theatre was erected in accordance with the requirements of Sir Victor Horsley and Mr. Ballance, it may be freely accepted that everything about it is satisfactory. The architect was Mr. R. Langton Cole, of Throgmorton Street.

## Figures and Tables

**Figure f1:**